# Hair Dye poisoning: “An early intervention”

**DOI:** 10.12669/pjms.341.14123

**Published:** 2018

**Authors:** Syed Farrukh Umair, Imrana Amin, Ata Urrehman

**Affiliations:** 1 Dr. Syed Farrukh Umair, FCPS. Department of Medicine, Patel Hospital, Karachi, Pakistan; 2 Dr. Imrana Amin, FCPS. Department of Medicine, Patel Hospital, Karachi, Pakistan; 3 Dr. Ata Urrehman, FCPS. Department of Medicine, Patel Hospital, Karachi, Pakistan

**Keywords:** Creatine phosphokinase (CPK), Paraphenylene diamine (PPD)

## Abstract

The use of hair dye has been emerging worldwide however usage of Paraphenylenediamine (PPD) in making hair dye is generally restricted to underdeveloped and developing countries. In particular, prevalence of accidental and suicidal ingestion is more in low socioeconomic areas. The spectra of hair dye toxicity is wide, however, it presents more commonly with severe angioedema of face and neck leading to respiratory failure, rhabdomyolysis complicating into acute kidney injury, myocarditis and acute liver injury. Here we present a unique case of PPD poisoning in a young female presented with laryngeal edema and marked rhabdomyolysis. Preemptive shifting to Critical care unit and elective endotracheal intubation for air way patency obviated the need of tracheostomy and precluded its related complications. Moreover, aggressive intravenous hydration prevented from renal failure despite markedly raised Creatine phospho kinase (CPK) levels.

## INTRODUCTION

Hair Dye Poisoning is highly uncommon in western world due to strict formulary regulations, however, it is emerging as a common means of suicide in developing world particularly Africa and Asia.^1^ Using Hair dye as a suicidal tool is rising in Southeast Asia especially India as reported mortality reaching up to 23.92% with treatment.^2^ In the absence of any antidote, only early recognition of symptoms along with timely management of the same is the key treatment option. Major toxic substance in hair dye is Paraphenylene Diamine (PPD) which after its consumption causes life threatening cervico-facial edema needing emergent endotracheal intubation or tracheostomy and severe rhabdomyolysis leading to renal failure.

In our region, Pakistan, incidents have been published mostly from Sindh and Punjab^3^-^6^ reporting the clinical presentation after hair dye poisoning. Hair dye powder is readily available and its widespread usage in this part of the world makes it imperative to understand the clinical implications of its toxicity whether suicidal or accidental. This is rather an under reported and under documented problem in Pakistan. We believe our case will help in creating awareness and importance of early recognition and aggressive management about this potentially lethal poisoning.

## CASE REPORT

A 21 year old female was brought to the emergency department (ED) in a tertiary care facility with shortness of breath and stridor. She took about 150 ml-200ml of hair dye, six hours prior to presentation with suicidal intentions. On examination her pulse rate was 105 beats /minute, respiratory rate of 38 breaths/minute, blood pressure of 148/90 mmHg, with SpO2 of 95% at room air. She had a facial swelling extending up to the neck with mild stridor. Immediate Naso-gastric lavage was done and ENT consult was taken.

**Fig. 1:**
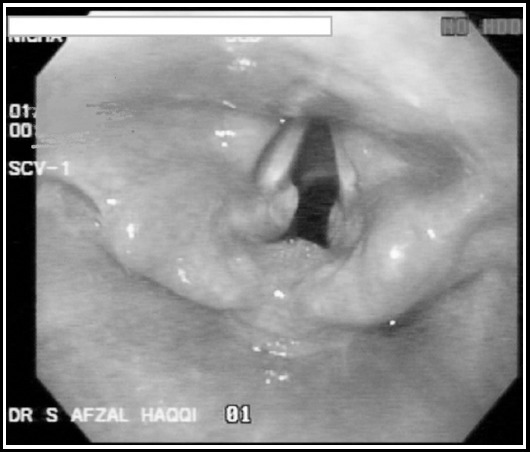
FibreOptic laryngoscopy of Patient after ingestion of hair dye, showing edematous epiglottis and larynx.

Bed side Fiber Optic Laryngoscopy (FOL) was performed in ED, which showed significant laryngeal edema Fig.1. Initial work up showed Hb 12.6 gm/dl TLC 8.7, Platelets 230,00, BUN 15 mg/dl creatinine 1.1 mg/dl, SGPT74 mg/dl and markedly raised CPK 54,023 IU/L depicted in Table-I. Patient was shifted to the monitoring area for observation.

**Table-I:** Hematological and Biochemical Parameters of Patient on Day-1 till day-3.

	Day-1	Day-2	Day-3
Hemoglobin	12.6 mg/dl		
Tlc	8.7		
platelets	230		
BUN/ Creatinine	16/1.0	18/0.9	15/0.8
SGPT	74		
CPK	54,023	67,021	97,628

Twelve Hours after admission, patients respiratory distress was worsening slowly with facial plethora and increase in stridor. Critical care team decided to electively intubate the patient, after family consent, for air way patency to avoid the risk and complications of emergency tracheostomy. Day-2 and 3, her condition deteriorated in terms of cervico-facial edema and CPK level mounted up to 95,041 IU/L.

Central venous pressure guided intravenous hydration at a rate of 200ml/hour plus, bicarbonate infusion at a rate of 150 ml/hour for urine alkalization was continued. Intravenous dexamethasone 4 mg every 8 hourly was started from day-1 to reduce cervico-facial edema. Laryngeal edema was accessed daily via cuff-leak pressure test. Patient's renal functions remain stable as shown in Table-I, maintaining good urine output. Day-4, she showed signs of improvement in laryngeal edema and CPK levels started waning off.

Day-5, patient was successfully extubated following weaning protocol. Vocal responses progressively improved subsequently. Upon shifting to Ward, meeting with in-house Psychiatrist was arranged. Patient was discharged on Day-7 with no residual deficit.

## DISCUSSION

One of the cheapest forms of chemical used for making hair dye and Henna is taken from black stone regionally known as *“kalapathar”*. It contains a toxic compound Para Phenyline Diamine (PPD) having highly toxic properties. The first ever toxicity of PPD was identified after occupational exposure by hair dresser in1924.^7^ The consequences are life threatening as it affects almost all body systems.^2^ It is an aromatic amine, a coal-tar derivative, which after oxidation converts into Brandowaski's base, un-natural and widely produced by commercial Industries in textile, fur dyes, cosmetics and gasoline.^8^

If not all, most cases of hair dye poisoning occur with suicidal intentions more common in young age females.^9^ It is gaining popularity due to easy availability and low cost. The prevalence of PPD toxicity has been variably documented more in low socio-economic regions like South Asia and Africa.^10^ Whereas, it's abuse as self-harm substances is much less reported in well developed countries like Europe and America due to strict industrial formulary regulations.

High proportions of cases are identified as oral ingestion however trans-dermal toxicity has been reported as well.^11^ Ingestion causes systemic toxicity^12^ in dose dependent manner^13^ even as little as 25 ml of oral ingestion causing hepatitis.^13^ PPD toxicity presents with two principal features; Firstly, cervical Orofacial angioedema and dysphagia due to local mucosal irritation as well as systemic allergic reaction frequently requiring emergency tracheostomy, starting within 6-8 hours in 80% of cases as stated by Kallel et al.^11^,^14^,^15^ It is usually followed by rhabdomyolysis and acute renal failure adding significantly to morbidity and mortality reported as high as 23.92% from India^2^,^16^ other systemic features include myocarditis involving ventricular tachyarrhythmia^17^ and high leukocyte count.

In the present case, only three clinical features manifested that is angioneurotic edema, rhabdomyolysis and tachycardia. However early recognition of symptoms and clear history tipped us to intervene early for elective intubation and escape emergent tracheostomy later. Especially after elective intubation, aggressive fluid resuscitation without the fear of volume overload helped us preventing expected renal failure. A case series from India reports higher mortality 27% in anuric patients.^9^ Acute kidney Injury ranges from benign proteinuria for a week to direct toxic insult to tubules leading to reversible acute tubular necrosis (ATN).^16^ PPD is not dialyzable and no specific antidote available, hence early recognition and supportive care has a major role in preventing catastrophic events. Hydrocortisone and anti-histamine have been used to reduce oro-facial edema.

## CONCLUSION

Hair dye poisoning is very common in Pakistan. It is a cost effective alternative to Organophosphate poisoning and is readily available to masses. Cervico-facial edema requiring tracheostomy and rhabdomyolysis leading to acute kidney Injury are main cause of morbidity and mortality. Since laryngeal edema is reversible and settles down in a week, early Endo-tracheal intubation can preclude tracheostomy which can hasten recovery with much shorter hospital stay. More case series are required to confirm effectiveness of early intubation as an alternative to tracheostomy as a part of management.

### Author`s Contribution

**SFU** identified and prepared the manuscript and take full responsibility for its clinical integrity.

**IA** helped in preparing Abstract and writing discussion. She also reviewed the final manuscript.

**AR** helped in abstract and discussion writing.
